# Characterization of Simple Sequence Repeat (SSR) Markers Mined in Whole Grape Genomes

**DOI:** 10.3390/genes14030663

**Published:** 2023-03-07

**Authors:** Dan Pei, Siyan Song, Jun Kang, Chuan Zhang, Jing Wang, Tianyu Dong, Mengqing Ge, Tariq Pervaiz, Peian Zhang, Jinggui Fang

**Affiliations:** 1 Fruit Crop Genetic Improvement and Seedling Propagation Engineering Center of Jiangsu Province, College of Horticulture, Nanjing Agricultural University, Nanjing 210095, China; 2 Zhenjiang Institute of Agricultural Sciences in Hilly Area of Jiangsu Province, Zhenjiang 212400, China; 3 Department of Botany and Plant Sciences, University of California Riverside, Riverside, CA 22963, USA

**Keywords:** grape, cultivated species, SSR, motif features, variety evolutionary tree

## Abstract

SSR (simple sequence repeat) DNA markers are widely used for genotype DNA identification, QTL mapping, and analyzing genetic biodiversity. However, SSRs in grapes are still in their early stages, with a few primer pairs accessible. With the whole-genome sequencing (WGS) of several grape varieties, characterization of grape SSR changed to be necessary not only to genomics but to also help SSR development and utility. Based on this, we identified the whole-genome SSR of nine grape cultivars (‘PN40024’, ‘Cabernet Sauvignon’, ‘Carménère’, ‘Chardonnay’, ‘Merlot’, ‘Riesling’, ‘Zinfandel’, ‘Shine Muscat’, and ‘Muscat Hamburg’) with whole-genome sequences released publicly and found that there are great differences in the distribution of SSR loci in different varieties. According to the difference in genome size, the number of SSRs ranged from 267,385 (Cabernet Sauvignon) to 627,429 (Carménère), the density of the SSR locus in the genome of nine cultivars was generally 1 per Kb. SSR motif distribution characteristic analysis of these grape cultivars showed that the distribution patterns among grape cultivars were conservative, mainly enriched in A/T. However, there are some differences in motif types (especially tetranucleotides, pentanucleotides, and hexanucleotides), quantity, total length, and average length in different varieties, which might be related to the size of the assembled genome or the specificity of variety domestication. The distribution characteristics of SSRs were revealed by whole-genome analysis of simple repeats of grape varieties. In this study, 32 pairs of primers with lower polymorphism have been screened, which provided an important research foundation for the development of molecular markers of grape variety identification and the construction of linkage maps of important agronomic traits for crop improvement.

## 1. Introduction

Molecular markers that reveal polymorphism at the DNA level have proved to be a very powerful tool for characterizing and estimating genetic diversity [1,2]. The fast expansion of molecular markers has been fostered by the advancement of genetic research and the lowering of genotyping costs in recent years [3]. At present, different genetic marker assistance systems have been developed [4]. However, compared with other DNA markers, microsatellites or SSR markers have become valuable molecular tools for genetic fingerprint analysis because of their richness, high polymorphism, co-dominant inheritance, high repeatability, and suitability for automation [5,6,7]. Furthermore, they are very variable, and the associated data can be easily compared between laboratories, and basic laboratory equipment suitable for small- and medium-sized laboratories is required [8].

SSR markers have 2–6 bp tandem repeats in the core sequence, which are highly repeatable, and rich in the genome, and the number of repeats at specific sites is highly variable. Most SSR markers used in grape variety identification are generated by cross-amplification in the development of expressed sequence tag (EST)—SSR primer pairs [9,10,11,12]. Although EST-SSR can be used for genetic analysis and has been widely used to estimate genetic diversity and construct linkage maps, its relatively high sequence conservation and high-concentration gene-rich regions with low polymorphism levels may limit its application, especially in the construction of a linkage map [13]. In contrast, genomic SSR markers are highly polymorphic and tend to be widely distributed throughout the genome, resulting in better map coverage [14,15]. However, in distant species, the conservation of genomic SSR loci is low, so their applicability decreases with the increase in genetic distance [16,17]. Therefore, the development of a large number of original SSR markers will contribute to the genetic and breeding research of species. Next-generation sequencing technology stands out because of its low price of analysis and high performance of data acquisition, which has become a new tool for detecting molecular markers [18,19].

Since the first time, scientists have effectively translated the genetic code of a fruit plant in 2007, considerably advancing grape functional genomics and grape molecular biology research [20]. With the rapid development of sequencing technology, more and more important grape varieties have been sequenced [21]. A large number of molecular markers are developed based on the level of the grape genome, which can be applied to variety identification, genetic diversity research, population structure analysis, association mapping, and linkage mapping [22,23,24]. All these applications support the increase in grape basic genetics research and breeding activities. The effectiveness of SSR in grapes plays a fundamental role in the rapid genetic separation of complex traits, such as resistance to biological and abiotic stresses and the identification of quantitative trait locus of various important traits [25]. For better utilization of grape SSR, here, we used large-scale genome sequence data to characterize SSR in several grape genomes.

## 2. Experiment Method and Content

### 2.1. Plant Materials

A collection of eight random grape cultivars (‘Hongxiangjiao’, ‘Jumeigui’, ‘Pione’, ‘Kuroshio’, ‘Jingzijing’, ‘Queen of Vinyard’, ‘Pinot Noir’, and ‘Cabernet Sauvignon’) were obtained from the national grape germplasm repository of Zhengzhou Fruit Research Institute at Chinese Academy of Agricultural Sciences. Those samples were used to validate the usefulness of SSR primer pairs. The DNA was extracted using the hexadecyl trimethyl ammonium bromide (CTAB) method [26].

### 2.2. Data Sources

In this study, nine *Vitis* materials that have completed genome sequencing were selected. There were seven *Vitis vinifera* ssp. *vinifera* (taxonomy id: 29760) germplasm resources, ‘Cabernet Sauvignon’ [27,28,29], ‘Carménère’ [30], ‘Chardonnay’ [31], ‘Merlot’ [32], ‘Riesling’ [33], ‘Zinfandel’ [34], and ‘Pinot Noir’ (‘PN40024’) [20,35,36]; and two *Vitis labruscana* × *Vitis vinifera* (taxonomy id: 2817680) resources, ‘Shine Muscat’ [37] and ‘Muscat Hamburg’ (sequencing sequence not published).

Except for *V. labruscana* × *V. vinifera* resources (‘Shine Muscat’ and ‘Muscat Hamburg’), the genome sequences of other grape cultivars are mainly downloaded from http://www.grapegenomics.com/pages/all.php (accessed on 15 December 2021).

### 2.3. Identification of SSR Motifs

SSRs of nine grape cultivars were identified by the microsatellite identification tool. A Perl5 script called MISA (http://pg-rc.ipk-gatersleben.de/misa/misa.html (accessed on 3 January 2022)) was used to identify and locate perfect SSRs and composite SSRs interrupted by a certain number of bases. The repeat unit length is defined as the default mononucleotide to hexanucleotide, as microsatellites with long repeat units are very rare. For mononucleotide to hexanucleotide, the minimum repeat units are defined as 10, 6, 5, 5, 5, and 5, respectively. Composite microsatellites are defined as repeats with ≥2 repeats interrupted by ≤100 bp [38]. Primer 3 [39] was used to design primers for sequences containing SSR loci. Primers were designed by using the genomic sequence of ‘PN40024’. The parameters for primers are: length: 18–25 bp, GC content: 40–60%, annealing temperature (TM): 55–60 °C, and desired amplification product size: 100–500 bp. Eighty pairs of primers were randomly selected and sent to General Biol Co., Ltd. for synthesis. PCR products were detected by nondenaturing polyacrylamide gel electrophoresis (PAGE) [40]. SSR primers were initially standardized for optimum annealing temperature at different gradient temperatures. The PCR procedure was 94 °C for 3 min, then 29 cycles at 94 °C for 30 s, 55 °C for 30 s, 72 °C for 45 s, and a final extension at 72 °C for 10 min. The number of alleles and polymorphism information content (PIC) for the SSRs were calculated using Anderson’s method [41].

### 2.4. Distribution Density of SSRs and SNPs in the Genome

To investigate the motif distribution of SSRs and single-nucleotide polymorphism (SNP) density in the genome of ‘PN40024’ 19 chromosomes (chr1–chr19), we have used Circos (v 0.69) [42]. This tool is effective in displaying structure variation in genomes. In this paper, a circle figure can be created from SSRs/SNPs and karyotype data inputs.

### 2.5. Functional Annotation of SSR Loci

We used SnpEff 5.0e software (https://pcingola.github.io/SnpEff (accessed on 3 June 2022)) for gene annotation of SSR loci. In this paper, SSR loci results can be created from genome gtf and SSR data inputs.

### 2.6. Phylogenetic Analysis

SNPhylo software (https://chibba.pgml.uga.edu/snphylo/ (accessed on 18 January 2022)) [43] was used to construct a phylogenetic tree. SNPhylo accepts a vcf input, removes low-quality data, extracts SNPs in an approximate linkage equilibrium, aligns SNPs with MUSCLE, and generates a phylogenetic tree in the PHYLIP package [44]. A SNPhylo phylogenetic tree was generated by using the default parameters: linkage disequilibrium (LD) is 0.1, minor allele frequency (MAF) is 0.1 and the missing rate is 0.1.

## 3. Results

### 3.1. Number and Density of SSRs Identified in Nine Grape Genomes

The analysis indicated that the numbers of SSR on nine grape cultivars’ chromosomes were different, ranging from 297,659 in ‘PN40024’ to 627,429 in ‘Carménère’ (Table 1). When considering their assembled genome sizes, the SSR loci densities showed another variation, even though the lowest and the most were still in ‘PN40024’ and ‘Carménère’, respectively. Except for the relatively lower density of ‘Carménère’ (0.99), the others were all over 1 SSR loci per 1 Kb.

The distribution of the number of SSRs on chromosomes is shown in Table S1. It can be seen that, on the whole, the distributions of the number of SSRs on chromosomes of all varieties show the same regular patterns. Namely, there is the smallest quantitative distribution on chromosome 17 (accounting for 3.33–4.02%); the largest number of SSRs was distributed on chromosome 14 or 18 (accounting for 5.78–7.64%). The variation of the total number of SSRs on chromosome 3 among the nine cultivars was the smallest, only 9.16%, while the variation on chromosome 13 was the largest, reaching 31.89%. Except for hexanucleotides that had the smallest variation in the number distribution of chromosome 10, the number distribution variation of other SSR motif types on chromosome 3 is the smallest, only 8.48–10.50%. Pentanucleotides and hexanucleotides had the largest variation in the number distribution of chromosomes 13 and 4, reaching 35.60% and 51.09%, respectively.

Among different varieties, the distribution density of SSRs on chromosomes showed obvious differences (Table S2), and no obvious regularity was found. The SSR distribution densities on chromosomes 2, 17 and 19 were lower, while those on chromosomes 1, 9 and 16 were high. The variation of SSR distribution densities on chromosome 18 among cultivars was the lowest, only 1.81%, while those on chromosome 19 were the largest, reaching 5.00%. In general, with the increase in the number of nucleotides, the distribution density variation coefficient of SSRs on chromosomes showed a gradually increasing trend. That is, the coefficient of variation of the distribution density of a single nucleotide on the chromosome is the smallest, only 0.90%, while the distribution density variation coefficient of hexanucleotides on the chromosome was the largest, reaching 8.47%.

**Figure 1:**
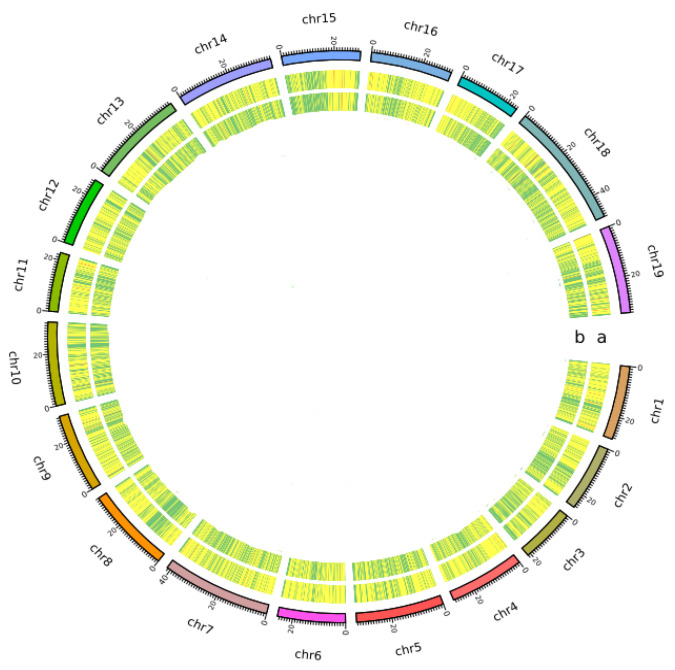
Overall view of the motif distribution of SSRs and SNP density in the genome of nineteen ‘PN40024’ chromosomes (chr1–chr19) was assembled by Hi-C. (**a**) SSR; (**b**) SNP.

**Figure 2:**
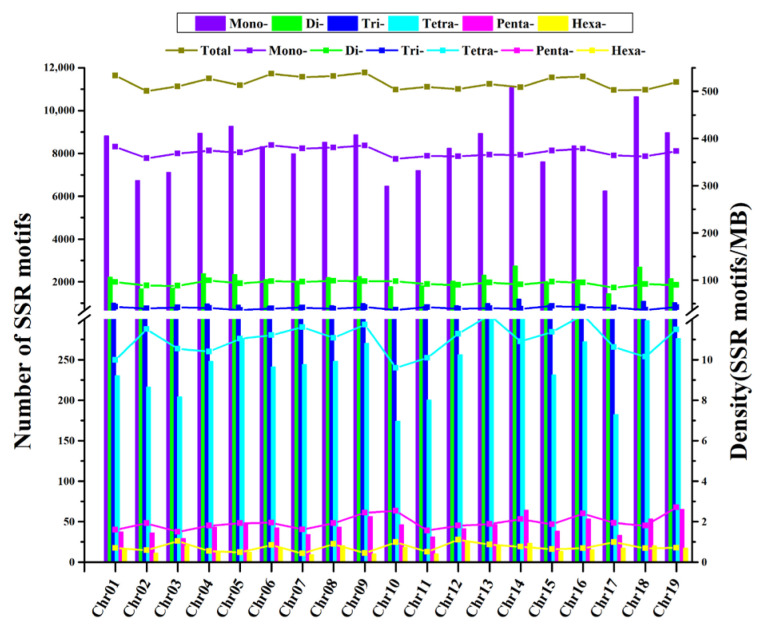
Number and density distribution of SSRs on 19 chromosomes in the genome of cv. ‘PN40024’. The bar graph shows the number of SSR loci. The linear graph shows the density of SSR loci. The horizontal ordinate is chromosome number, vertical coordinates are number of SSR motifs and SSR motif density, respectively.

The distribution of SSRs in the grape genome was presented using the number and density distribution of ‘PN40024’-derived chromosomal SSRs as an example. The overall view of the motif distribution of SSRs and SNP density in the genome of ‘PN40024’ 19 chromosomes (chr1–chr19) was assembled by Hi-C as shown in Figure 1. It can be seen that there is no significant difference between the distribution uniformity of SSR and SNP on 19 chromosomes. The SSR motifs in the genome of the variety ‘PN40024’ and the distribution quantity and density on different chromosomes of SSR are shown in Figure 2. It can be seen that the distribution quantity and density of SSR motifs on different chromosomes are different. Chromosome 9 has the largest distribution density, reaching 539.63/Mb; the minimum distribution density of chromosome 2 was only 500.96/Mb; the distribution density of SSR motifs on other staining ranged from 502.54/Mb (chr17) to 537.46/Mb (chr6). In addition, the number of SSR motifs on each chromosome (chr1–chr19) of ‘PN40024’ varies from 8607 (chr17) to 15,403 (chr14). In addition, we found a positive correlation between the number of SSR motifs on chromosomes and the genome assembly size (Figure S1), while there was no significant correlation between genome size and its distribution density (Figure S2).

### 3.2. Types of SSRs Repeat Motifs and Lengths in the Grape Genome

**Figure 3:**
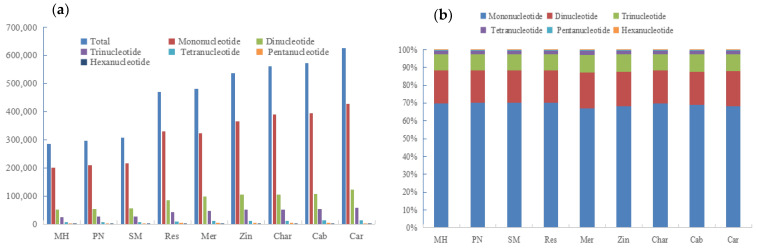
Distribution of SSR motif number (**a**) and proportion (**b**) in genome sequences of different grape varieties.

There are six different types of SSR repeat motif lengths, as illustrated in Figure 3, including mononucleotides, dinucleotides, trinucleotides, tetranucleotides, pentanucleotides, and hexanucleotides, with mononucleotide loci accounting for 70% below and upper of the total SSR loci on the nine genomes. It was discovered that the longer the repeat motifs, the lower the SSR locus numbers, with the hexanucleotide loci in ‘Chardonnay’ accounting for only 0.12% of the total locus number. It was also found that the repeat motif types are very different among the 6 kinds of SSR loci, and the longer the repeat motif, the more the repeat motif types, from 2 of mononucleotide loci to 82 of hexanucleotides (Table 2 and Table S3). Among them, there are all types of mononucleotides, dinucleotides, and trinucleotides in the nine grape genomes, while the existence of tetranucleotides, pentanucleotides, and hexanucleotides showed differences among the genomes. Most interestingly, each kind of length SSR loci has a dominant type of repeat motif with most A/T nucleotide pairs. For example, A/T is rich in mononucleotide loci, and AT/AT, AAT/ATT, AAAT/ATTT, AAAAT/ATTTT, and AAAAAT/ATTTTT are the main motif types of dinucleotides, trinucleotides, tetranucleotides, pentanucleotides, and hexanucleotides, respectively, covering 65.334–68.659%, 11.937–13.387%, 6.702–7.434%, 1.396–1.545%, 0.1680–0.2090%, and 0.0220–0.0320%.

### 3.3. Length of Various SSR Loci and Repeating Times of the Different Repeat Motifs

As shown in Table S4, it was found that duplicated times of all the six kinds of long repeat motifs were different, which determined the length of the corresponding locus. In total, the longer the motifs, the fewer the number of lower duplicating times. For example, the duplicating times of mononucleotide SSR loci were the highest ranging from 11 to 12 in the ‘PN40024’ genome, while that of the pentanucleotide loci was the lowest from 5 to 6. Those of dinucleotides, trinucleotides, tetranucleotides, and pentanucleotides were 6 to 7, 5 to 6, 5 to 6, and 5 to 6, respectively. The phenomena in the other eight genomes were similar (Table S4).

**Figure 4:**
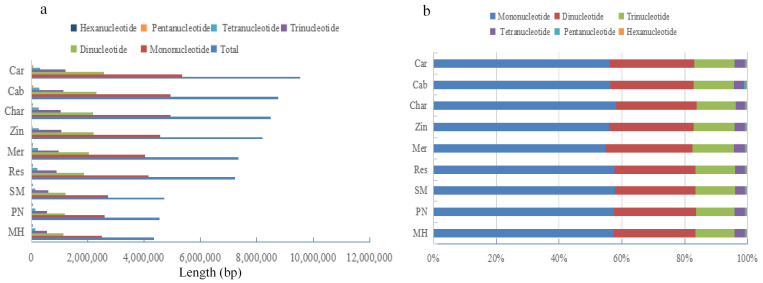
The overall lengths (**a**) and proportion (**b**) of microsatellites in genome sequences of different grape varieties.

The overall lengths of SSRs differed between and among the six types of motif length groupings (Figure 4). The lengths of mononucleotides, dinucleotides, trinucleotides, tetranucleotides, pentanucleotides, and hexanucleotides had different ranges of 200,117 bp–428,787 bp, 52,587 bp–122,412 bp, 25,857 bp–59,162 bp, 6138 bp–13,897 bp, 1053 bp–2305 bp, and 433 bp–921 bp., respectively. Among each motif length group, the SSRs with the motifs including AT/AT, AAT/ATT, AAAT/ATTT, AAAAT/ATTTT, and AAAAAT/ATTTTT had the longest lengths, suggesting that the motifs with more A/T duplicated much more. The situation was the same in all the nine genomes.

### 3.4. Annotation of SSR Loci

By searching the ‘Chardonnay’ genome annotation file (GFF) (Table S5), we found that among the 561,635 SSR loci identified, 316,874 SSR loci were located in or near the gene. There were 224,001 SSRs in the intergenic region, followed by the number of SSRs located in introns (53,959). There are 7536 SSRs in 5 ‘UTR or 3’ UTR. Only 1890 SSRs were located in the exon region of the coding gene (Table S5).

The distribution of different motif SSRs in the ‘Chardonnay’ genome is shown in Table 3. We found that the mononucleotide-annotated SSR sites were the most, reaching 227,078; the SSR sites annotated by hexanucleotide were the least, only 409. In each nucleotide motif type, trinucleotides and hexanucleotides were distributed in the exon region of the coding gene in a large number, reaching 1363 and 46, respectively, while the number of mononucleotide, dinucleotide, tetranucleotide and pentanucleotide types distributed in the exon region of the coding gene was relatively low—281, 186, 11, and 3 (1.49%), respectively. We also found that the distribution of SSRs in the genome of other varieties showed a similar pattern.

### 3.5. Comparison of the Phylogenetic Trees of Nine Grape Cultivars Constructed Using All the SSRs and SNPs

**Figure 5:**
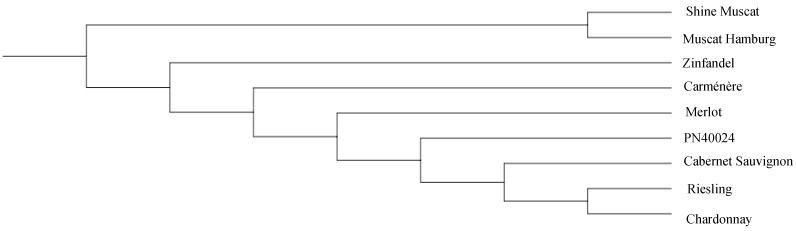
Construction of a grape variety evolutionary tree based on a simple repeat sequence.

**Figure 6:**
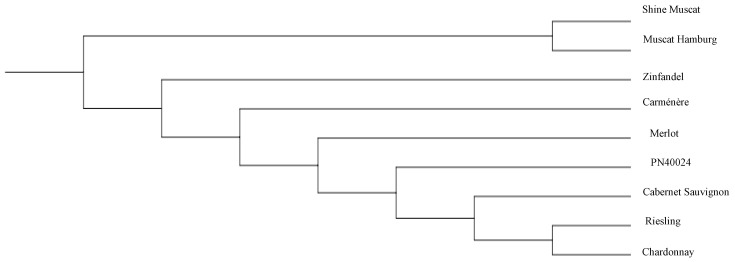
Construction of a grape variety evolutionary tree based on SNPs.

In order to evaluate the utility of the whole-genome SSR identified, both the SSRs and SNPs were utilized to analyze the phylogenetic trees of the nine grape cultivars. The result indicated that both the trees constructed using SSRs and SNPs were almost identical (Figure 5 and Figure 6). In general, the nine varieties that have been sequenced can be divided into two categories as shown in Figure 5 and Figure 6. One species is *Vitis vinifera* ssp. *vinifera*, with seven varieties, Cabernet Sauvignon’, ‘Carménère’, ‘Chardonnay’, ‘Merlot’, ‘Riesling’, ‘Zinfandel’, and ‘PN40024’derived. The other is *V. labruscana* × *V. vinifera*, with two varieties, ‘Shine Muscat ’and ‘Muscat Hamburg’. In addition, we found that ‘Riesling’ and ‘Chardonnay’ are closely related.

### 3.6. The Distribution and Numbers of ‘PN40024’ SSR Loci

**Figure 7:**
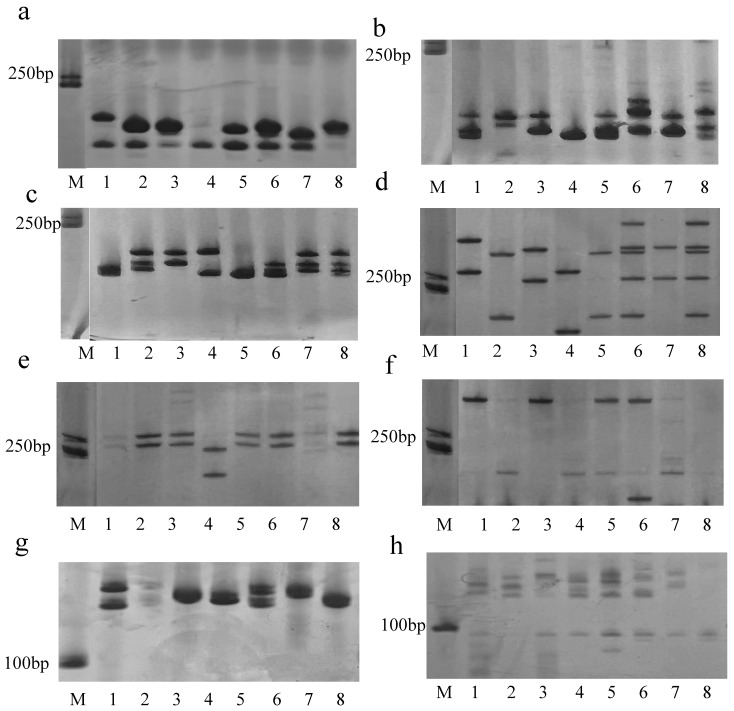
PCR amplification of SSR primers in eight random DNA. M: DNA ladder; sample 1-8. (**a**): 5-14; (**b**): 5-19; (**c**): 5-20; (**d**): 6-2; (**e**): 6-3; (**f**): 6-4; (**g**): 6-6; (**h**): 6-15.

In the ’PN40024’ genome data, 297,659 SSRs were identified, including 208,985 SSRs with mononucleotides, 54,196 SSRs with dinucleotides, 26,478 SSRs with trinucleotides, 6.438 SSRs with tetranucleotides, 1129 SSRs with pentanucleotides, 433 SSRs with hexanucleotides, and 50,927 compound SSRs. A total of 147,308 of them could be located on 19 chromosomes, accounting for 49% of the total SSRs. We mainly analyzed SSRs with 1–6 repeat units, and their distribution density on 19 chromosomes is shown in Figure 7, Figures S3 and S4.

Units 2 and 3 were the main types, with the size of repeat sequences more than 100 bp. On the contrary, long repeat units (5 and 6) failed to form long repetitive sequences. Primers were designed for randomly selected 80 SSR loci, and verified with 8 grape cultivars (Table S6). Among them, 68 pairs of primers were able to detect amplification products, of which 32 pairs (40%) of primers had relatively low polymorphism, which could be used to distinguish different varieties. Among the 32 pairs of primers that can provide low polymorphism, 25 pairs are from 5 and 6 SSR repeat units, indicating that the variation between long repeat units can be more reflected in different varieties. A total of 134 bands were generated in the amplification results of all type b primers, of which 113 (84.33%) could be used for variety discrimination (Table S6).

## 4. Discussion

With the rapid development of sequencing technology, many SSR loci can be found quickly and cost effectively by next-generation sequencing (NGS) technology [18,19,45,46]. Whole-genome-based SSR locus mining can be much more efficient and output higher numbers of SSR markers than the method of rapid isolation of repeat containing sequences (fiasco) by amplified fragment length polymorphism (AFLP) or the traditional magnetic bead enrichment method or library, etc. The discovery and mining of SSR loci through genome sequences have been successfully applied to many plant species, including field crops, cash crops and medicinal plants [47,48,49,50,51]. All these do much help in plant genotyping, QTL mapping, and analyzing biodiversity, etc. With grape whole-genome sequencing (WGS), the characterization of the SSR loci is necessary and important.

### 4.1. Relationship between the Grape Genome Sizes and the Distribution Density of SSR on Chromosomes

The present study analyzed the distribution of SSR (composed of 1–6 bp) in nine genomes of grape cultivated varieties. Except for ‘Riesling’, similar to plant species such as *Capsicum* [52] and *Cucurbitaceae* [51], the number of the SSR motifs in grapes was positively correlated with genome size (r^2^ = 0.92, Figure S1). In contrast, SSR density is usually negatively correlated with genome size [53], however, similar to *Cucurbitaceae* plants (r^2^ = 0.89, Figure S2) [51]. It is worth noting that, similar to the more compact genome of *Cucurbitaceae* [51], the genome of grape species also shows a similar trend.

The classification of SSR motifs in the grape crop genome was compared initially. Compared with ‘PN40024’, the ‘Cabernet Sauvignon’ genome has more motif types, which may be partly due to the larger genome assembly size of the latter [51]. In SSR motifs, the changes in repeat selectivity constraints may be different [54]. In general, A/T-rich motifs appear more frequently in di-cotyledons [52,55]. The theme forms of classification in each variation, however, are not the same. Cucurbitaceae has had similar non-identities documented [51] and *Capsicum* genomes [52]. In the grape genome, we found comparable results. In all nine genomes, A/T, AT/AT, and AAT/ATT are the top three abundant motifs. However, AC/GT and C/G content shows different modes among nine cultivars, which indicate SSR contains sufficient information to distinguish different varieties.

In addition, there was no significant difference in the distribution uniformity of SNPs and SSRs on the ‘PN40024’ genome; therefore, SSR has the potential to be an alternative approach for variety identification and correlation analysis.

### 4.2. Conservation and Variety Specificity of the SSR Distribution Pattern of Grape Cultivars

Although the genome assembly sizes of the nine grape varieties analyzed in this study are different (‘Cabernet Sauvignon’, 591.0 Mb; ‘Carménère’, 622.8 Mb; ‘Chardonnay’, 606.0 Mb; ‘Merlot’, 606.1 Mb; ‘Muscat Hamburg’, 457.0Mb; ‘PN40024’, 486.0 Mb;‘Riesling’, 741.8 Mb; ‘Shine Muscat’, 484.89 Mb; ‘Zinfandel’, 591.20 Mb), we found that the SSR distribution pattern was conservative in these grape varieties. Firstly, the nine grape cultivated varieties have the same mononucleotide, dinucleotide, and trinucleotide types, and the main motif types are A/T, AT/AT, and AAT/ATT. Although distinct kinds of tetranucleotides, pentanucleotides, and hexanucleotides show varying degrees of deletion, the fundamental nucleotide motif types are essentially the same. The evolutionary mechanism of generating and maintaining high-order repeats is conservative in grape genomes [56]. These results suggest that the characteristics of SSRs may be species dependent and may be related to their evolution and adaptation. In general, SSR distribution patterns are consistent with species differentiation [57]. The number and length of varieties are obviously different. The difference in motif length and frequency between genomic and gene SSRs is likely due to gene SSRs’ selection pressure, which limits mutation fixation, resulting in a frameshift. [18]. SSR is mainly caused by replication slip, resulting in the addition or deletion of repeat motifs in the plant genome. Therefore, specific numbers and lengths of SSRs may reflect the evolutionary history of specific species and their relatives [58].

### 4.3. Grape Genome Sequencing to Construct a Grape Variety Evolutionary Tree and Reveal Its Potential Genetic Relationship

Through an evolutionary tree constructed based on genome sequencing data, we found that Chardonnay and Riesling are closely related. These two varieties may have common ancestors, which is consistent with the results of previous studies [59]. DNA fingerprint analysis of ‘Chardonnay’ shows that one of the ancestors of this noble grape is the “civilian” of grape cultivation. Modern DNA fingerprinting studies at the University of California, Davis, show that Chardonnay is the result of hybridization between ‘PN40024’ and the rare ‘Gouais blanc’ (also known as ‘Heunisch Weiss’) grape variety. These two varieties are widely distributed in northeast France [59]. Riesling was once thought to be a wild vine native to the Rhine area, although there is little evidence to back up this statement. According to DNA fingerprinting, one of Riesling’s parents is ‘Gouais blanc’ [60]. Similar to ‘Gouais blanc’, ‘Kyoho’ and ‘Muscat Hamburg’ are related to many important varieties. Those varieties with good characters are applied to many breeding works. This has resulted in the narrow genetic relationship of grapes, and also created difficulties for variety identification. Therefore, a large number of SSR primers have been developed to help clarify the genetic relationship between them. Compared with the nine commonly used primers (VVS2, VVMD5, VVMD7, VVMD25, VVMD27, VVMD28, VVMD32, VZAG79 and VZAG62) (www.vivc.de (accessed on 20 March 2022)) and the SNP marker, genome-based SSR markers have the advantages of high precision and low price. These markers with more readable bands can be used for cultivar identification and traceability.

## 5. Conclusions

In this study, we identified 286,192 to 627,429 SSR motifs among nine grape genomes. Of these, the dominant SSR motif type in grapes is rich in A/T, AT/AT, AAT/ATT, and AAAT/ATTT while there are some differentiated motifs such as AC/GT and C/G. We then designed 80 SSR primer pairs and validated these by PCR amplification. Among them, there are 32 pairs of primers that can be used to assess the genetic diversity of grape samples. The SSR molecular markers discovered in this work will aid grape breeding, germplasm resource identification, and gene mining of critical agronomic features, all of which will assist speeding up the directional breeding process.

## Figures and Tables

**Table 1:** Total frequency of SSRs in grape cultivars.

Cultivars	SSRs Number	GC (%)	Genome Length (Mb)	Frequency	
				Per Mb	One Every (Kb)
‘Cabernet Sauvignon’	572,976	34.61	591.0	969.50	1.03
‘Carménère’	627,429	34.45	622.8	1007.43	0.99
‘Chardonnay’	561,635	34.34	606.0	926.79	1.08
‘Merlot’	481,491	34.56	606.1	794.41	1.26
‘Muscat Hamburg’	286,192	34.61	457.0	626.24	1.60
‘PN40024’	297,659	34.55	486.0	612.07	1.63
‘Riesling’	470,670	34.43	741.8	634.50	1.58
‘Shine Muscat’	308,365	34.49	484.9	635.94	1.57
‘Zinfandel’	537,606	34.42	591.2	909.35	1.10

**Table 2:** Analysis of repeat motif types in grape genome.

Repeat Type	Repeat Motif	Number	Proportion (%)	Frequency
Mononucleotide	A/T	196,103–419,278	65.334–68.659	0.4579–0.5442
	C/G	4014–9509	1.341–1.778	0.0134–0.0186
Dinucleotide	AT/AT	34,612–81,602	11.937–13.387	0.1666–0.1797
	AG/CT	13,551–30,551	4.5270–5.163	0.529–0.571
	AC/GT	4403–10,218	1.522–1.727	0.0167–0.0178
	CG/CG	21–50	0.0070–0.0100	0.001
Trinucleotide	AAT/ATT	19,253–44,482	6.702–7.434	0.0888–0.0953
	AAG/CTT	2884–6769	0.9700–1.1111	0.0113–0.0124
	ACC/GGT	702–1474	0.2300–0.2620	0.0025–0.0029
	AAC/GTT	631–1488	0.2120–0.2500	0.0024–0.0029
	AGG/CCT	509–1110	0.1610–0.1890	0.0019–0.0020
	ACT/AGT	179–418	0.0620–0.0770	0.0008–0.0010
	CCG/CGG	70–170	0.0240–0.0310	0.0002–0.0003
	ACG/CGT	62–143	0.0200–0.0240	0.0002
Tetranucleotide	AAAT/ATTT	4156–9370	1.396–1.545	0.0187–0.0200
	AATT/AATT	475–1100	0.1600–0.1810	0.0021–0.0022
	AAAG/CTTT	429–969	0.1450–0.1710	0.0021–0.0022
	ACAT/ATGT	321–743	0.1100–0.1300	0.0018–0.0020
	AAAC/GTTT	198–437	0.0640–0.0750	0.0008–0.0010
	AGAT/ATCT	146–368	0.0490–0.0600	0.0008–0.0009
	AGGG/CCCT	88–209	0.0260–0.0360	0.0004–0.0005
Pentanucleotide	AAAAT/ATTTT	530–1169	0.1680–0.2090	0.0027–0.0034
	AAATT/AATTT	48–112	0.0120–0.0180	0.0002–0.0003
	AATAT/ATATT	41–98	0.0140–0.0180	0.0003
	AAAAC/GTTTT	37–84	0.0100–0.0160	0.0001–0.0002
	CCCCG/CGGGG	30–53	0.0070–0.0120	0.0001–0.0002
	AAAGG/CCTTT	15–37	0.0040–0.0070	0.0001
Hexanucleotide	AAAAAT/ATTTTT	77–170	0.0220–0.0320	0.0004–0.0006
	AAAAAG/CTTTTT	75–174	0.0190–0.0300	0.0004–0.0006
	AGAGGG/CCCTCT	22–58	0.0060–0.0100	0.0001–0.0002
	AAAAAC/GTTTTT	13–32	0.0040–0.0060	0.0001
	ACTCCC/AGTGGG	7–28	0.0020–0.0050	0.0000–0.0001
	AAAAGG/CCTTTT	5–20	0.0020–0.0030	0.0000–0.0001

**Table 3:** Distribution of SSR motif types in the ‘Chardonnay’ genome.

		Mono-	Di-	Tri-	Tetra-	Penta-	Hexa-
Total number		227,078	56,388	25,230	6679	1090	409
Between genes		160,947	39,104	17,937	4973	790	250
Within genes	Exon	281	186	1363	11	3	46
	Others	44,466	11,970	3670	1107	199	83
Others		21,384	5128	2260	588	98	30

## Data Availability

Not applicable.

## Supplementary Materials

The following supporting information can be downloaded at: https://www.mdpi.com/article/10.3390/genes14030663/s1, Figure S1: Relationship between the number of SSRs in grape genome and the size of genome assembly; Figure S2: Relationship between grape genome size and SSR distribution density; Figure S3: PCR amplification of SSR primer in eight random DNA. M: DL2000; Sample 1 8. a: 1 3; b: 1 4; c 1 5; d: 1 6; e: 1 10; f: 2 4; g: 3 1; h: 3 2; i: 3 7; j: 3 8; k: 3 9; l: 4 2; Figure S4: PCR amplification of SSR primer in eight random DNA. M: DNA Ladder; Sample 1-8. a: 4-3; b: 4-4; c:4-6; d: 4-8; e: 4-9; f: 5-3; g: 5-4; h: 5-5; i: 5-7; j: 5-10; k: 5-12; l: 5-16; Table S1: The distribution of the number of microsatellites on chromosomes; Table S2: The distribution density of microsatellites on chromosomes; Table S3: SSR type and number of nine cultivars; Table S4: The repeating times of the different repeat motifs in different cultivars; Table S5: The genome annotation of Chardonnay; Table S6: Validation of SSR motifs and polymorphisms for 80 primer pairs.
