# Supplementary material for: Characterization of Simple Sequence Repeat (SSR) Markers Mined in Whole Grape Genomes
Source: Genes (Basel). 2023 Mar 7;14(3):663. doi: 10.3390/genes14030663 (PMC10048371; doi:10.3390/genes14030663)
Supplement: Supplementary file 1 [file genes-14-00663-s001.zip › supplement fig.pptx]

## Slide 1
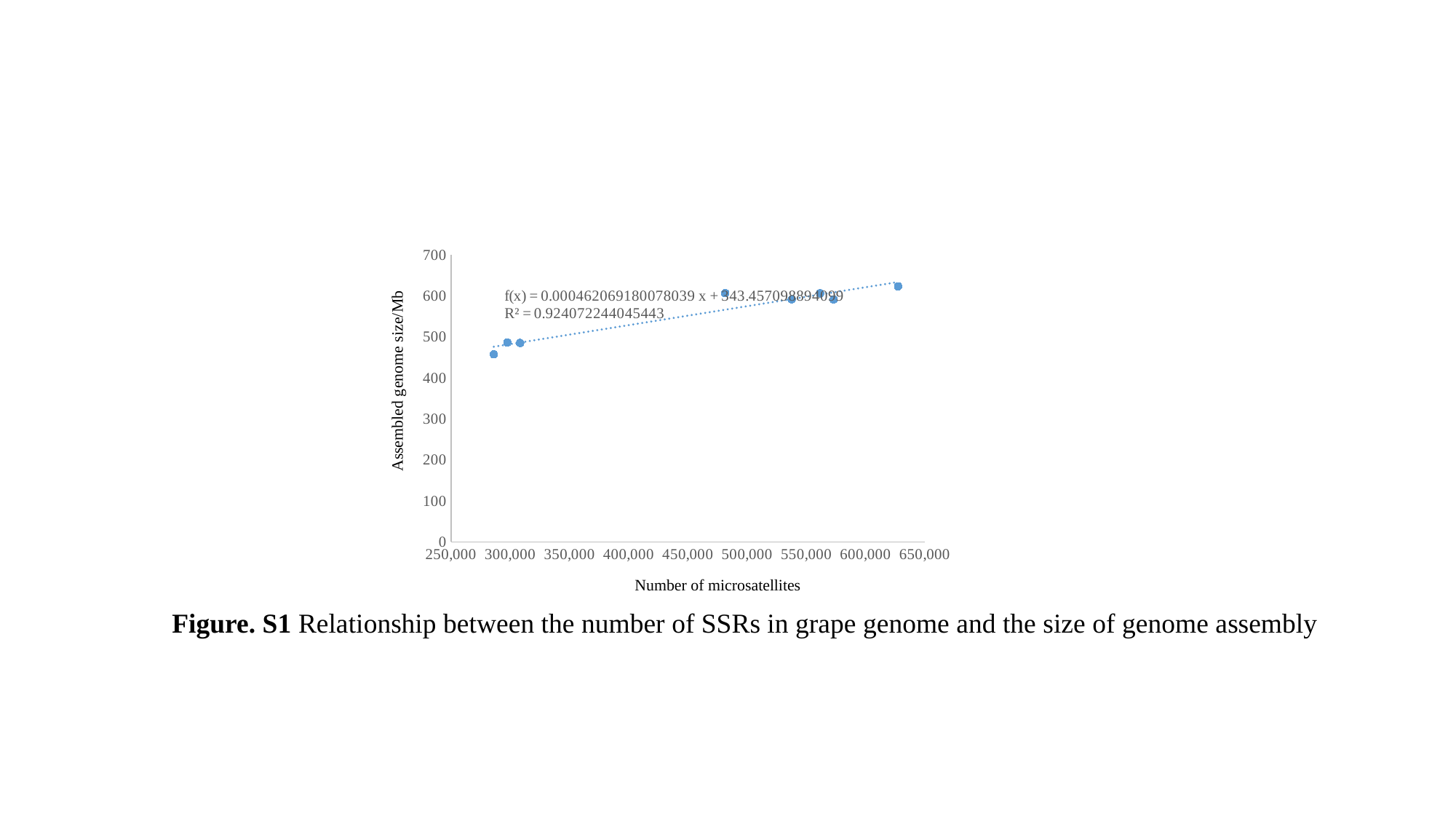

### Chart
| Category | Genome length (Mb) |
|---|---|Assembled genome size/Mb
Number of microsatellites
Figure. S1 Relationship between the number of SSRs in grape genome and the size of genome assembly

## Slide 2
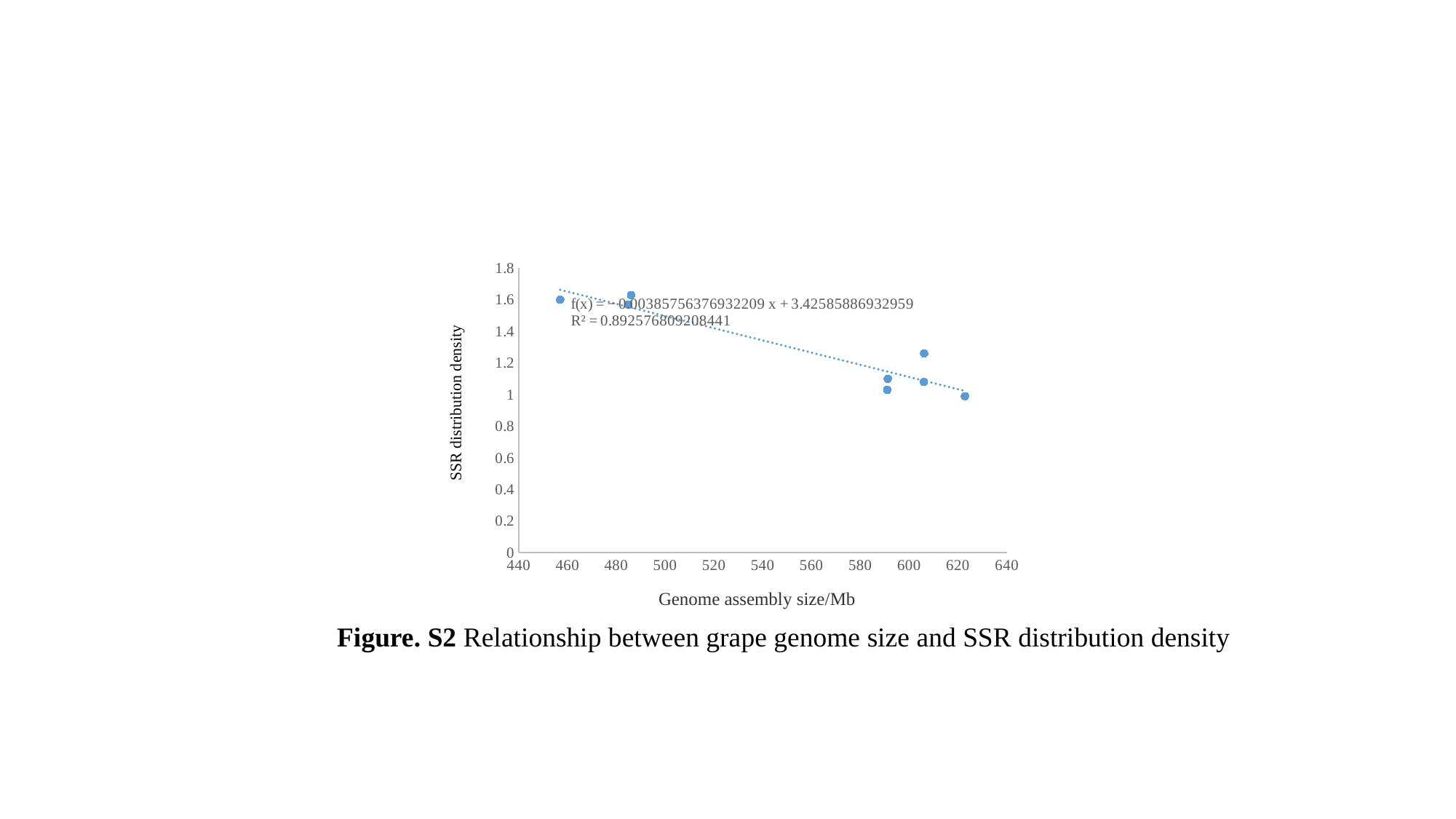

### Chart
| Category | Per Mb |
|---|---|SSR distribution density
Genome assembly size/Mb
Figure. S2 Relationship between grape genome size and SSR distribution density

## Slide 3
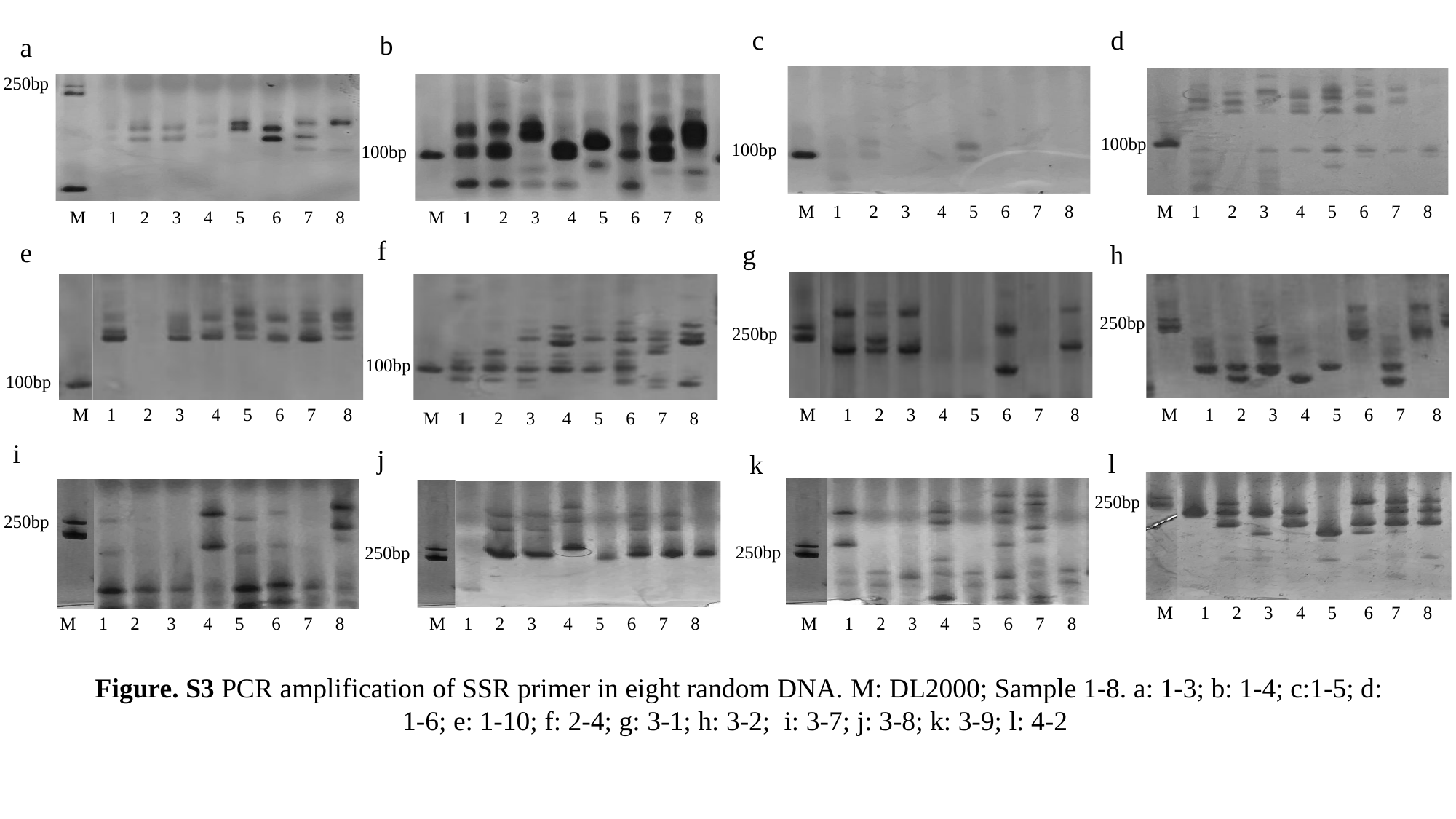

c
100bp
M 1 2 3 4 5 6 7 8
d
100bp
M 1 2 3 4 5 6 7 8
b
100bp
M 1 2 3 4 5 6 7 8
a
250bp
M 1 2 3 4 5 6 7 8
f
100bp
M 1 2 3 4 5 6 7 8
e
100bp
M 1 2 3 4 5 6 7 8
g
250bp
M 1 2 3 4 5 6 7 8
h
250bp
M 1 2 3 4 5 6 7 8
i
250bp
M 1 2 3 4 5 6 7 8
j
250bp
M 1 2 3 4 5 6 7 8
l
250bp
M 1 2 3 4 5 6 7 8
k
250bp
M 1 2 3 4 5 6 7 8
Figure. S3 PCR amplification of SSR primer in eight random DNA. M: DL2000; Sample 1-8. a: 1-3; b: 1-4; c:1-5; d: 1-6; e: 1-10; f: 2-4; g: 3-1; h: 3-2; i: 3-7; j: 3-8; k: 3-9; l: 4-2

## Slide 4
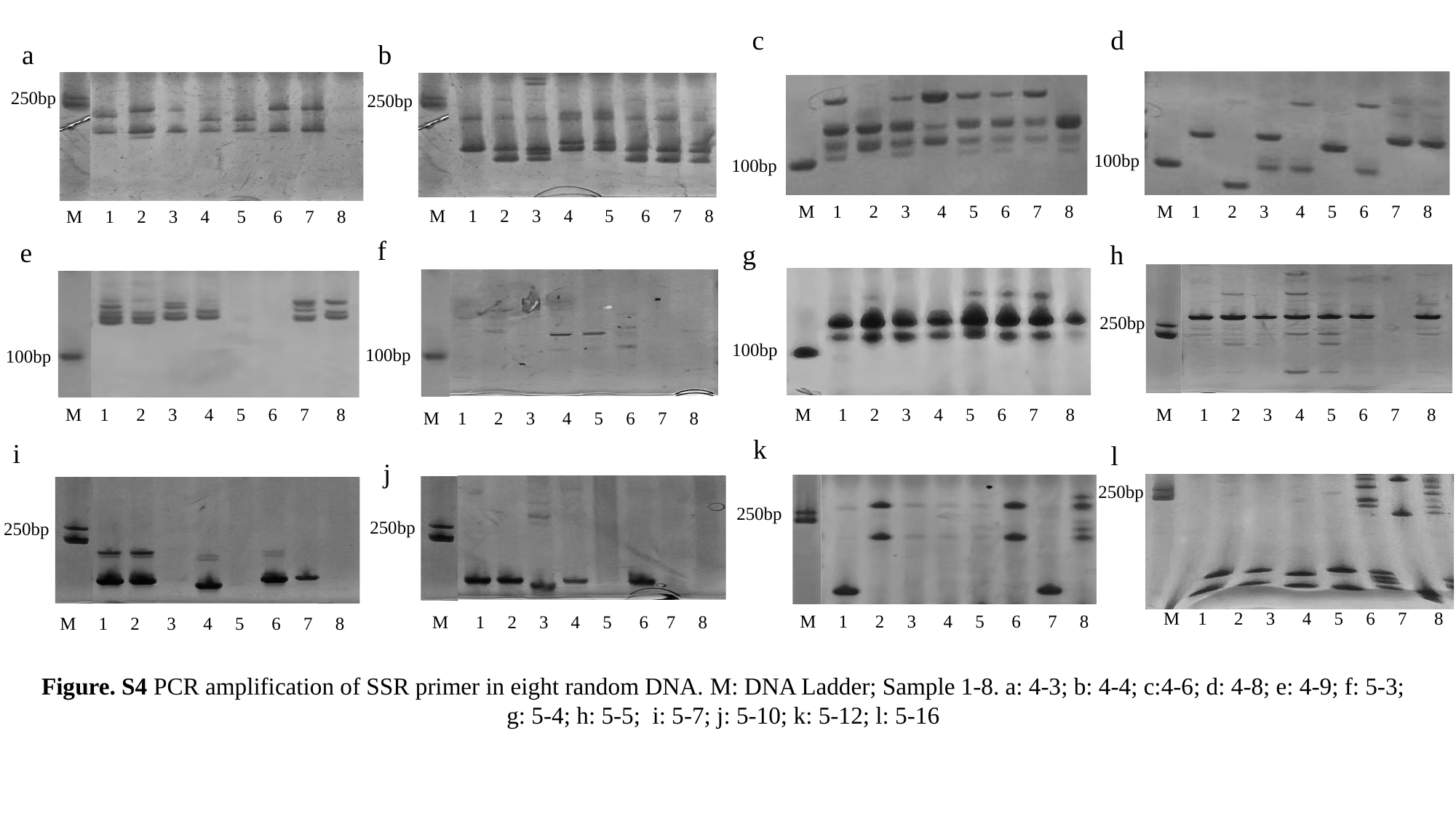

c
100bp
M 1 2 3 4 5 6 7 8
d
100bp
M 1 2 3 4 5 6 7 8
a
250bp
M 1 2 3 4 5 6 7 8
b
250bp
M 1 2 3 4 5 6 7 8
f
100bp
M 1 2 3 4 5 6 7 8
e
100bp
M 1 2 3 4 5 6 7 8
g
100bp
M 1 2 3 4 5 6 7 8
h
250bp
M 1 2 3 4 5 6 7 8
k
250bp
M 1 2 3 4 5 6 7 8
i
250bp
M 1 2 3 4 5 6 7 8
l
250bp
M 1 2 3 4 5 6 7 8
j
250bp
M 1 2 3 4 5 6 7 8
Figure. S4 PCR amplification of SSR primer in eight random DNA. M: DNA Ladder; Sample 1-8. a: 4-3; b: 4-4; c:4-6; d: 4-8; e: 4-9; f: 5-3; g: 5-4; h: 5-5; i: 5-7; j: 5-10; k: 5-12; l: 5-16
